# Geo-spatial Hotspots of Hemorrhagic Fever with Renal Syndrome and Genetic Characterization of Seoul Variants in Beijing, China

**DOI:** 10.1371/journal.pntd.0000945

**Published:** 2011-01-11

**Authors:** Shu-Qing Zuo, Li-Qun Fang, Lin Zhan, Pan-He Zhang, Jia-Fu Jiang, Li-Ping Wang, Jia-Qi Ma, Bing-Cai Wang, Ri-Min Wang, Xiao-Ming Wu, Hong Yang, Zhi-Wei Cao, Wu-Chun Cao

**Affiliations:** 1 State Key Laboratory of Pathogen and Biosecurity, Beijing Institute of Microbiology and Epidemiology, Beijing, China; 2 Centers for Public Health Information, Center for Disease Control and Prevention, Beijing, China; 3 Beijing Haidian Centers for Disease Control and Prevention, Beijing, China; 4 Beijing Dongcheng Centers for Disease Control and Prevention, Beijing, China; Tulane School of Public Health and Tropical Medicine, United States of America

## Abstract

**Background:**

Hemorrhagic fever with renal syndrome (HFRS) is highly endemic in mainland China, and has extended from rural areas to cities recently. Beijing metropolis is a novel affected region, where the HFRS incidence seems to be diverse from place to place.

**Methodology/Principal Findings:**

The spatial scan analysis based on geographical information system (GIS) identified three geo-spatial “hotspots” of HFRS in Beijing when the passive surveillance data from 2004 to 2006 were used. The Relative Risk (RR) of the three “hotspots” was 5.45, 3.57 and 3.30, respectively. The Phylogenetic analysis based on entire coding region sequence of S segment and partial L segment sequence of Seoul virus (SEOV) revealed that the SEOV strains circulating in Beijing could be classified into at least three lineages regardless of their host origins. Two potential recombination events that happened in lineage #1 were detected and supported by comparative phylogenetic analysis. The SEOV strains in different lineages and strains with distinct special amino acid substitutions for N protein were partially associated with different spatial clustered areas of HFRS.

**Conclusion/Significance:**

Hotspots of HFRS were found in Beijing, a novel endemic region, where intervention should be enhanced. Our data suggested that the genetic variation and recombination of SEOV strains was related to the high risk areas of HFRS, which merited further investigation.

## Author Summary

Hemorrhagic fever with renal syndrome (HFRS) is caused by Hantaviruses, the enzootic viruses with a worldwide distribution. In China, HFRS is a significant public health problem with more than 10,000 human cases reported annually and the endemic areas of the disease have extended from rural to urban areas and even to central cities in recent years. The HFRS incidence has increased recently and the morbidity seemed to be considerably diverse in different areas in Beijing, the capital of China. With the aim of gaining more information to control this disease, we carried out a spatial analysis of HFRS based on the data from human cases during 2004–2006 and investigated the genetic features of complete S and partial L segment sequences of Seoul virus from natural infected rodent hosts and patients. We found three geo-spatial clusters, i.e., “hotspots” of HFRS in Beijing, where intervention should be enhanced. Our data indicated that the genetic variation and recombination of SEOV might be related to the high risk areas of HFRS in Beijing, which was worthy of further investigation.

## Introduction

Hantaviruses are rodent-borne pathogens with a worldwide distribution. More than 50 hantaviruses have been found in the world [1]–[3], each of which appears to have coevolved with a specific rodent or insectivore host [4]. As with other members of the family *Bunyaviridae*, hantaviruses are enveloped, negative-sense RNA viruses. The genome consists of three segments, designated as large (L), medium (M), and small (S). They respectively encoded the RNA polymerase, the glycoprotein precursor (GPC) protein that is processed into 2 separate envelope glycoproteins (Gn and Gc), and the nucleocapsid (N) protein [1]–[4]. As nucleoproteins of many negative-strand RNA viruses, hantaviral N protein is a multifunctional molecule involved in various interactions during the life cycle of the virus. It has important functions in the viral RNA replication, encapsidation and also in the virus assembly [5].

Hantavirus can cause two kinds of human illnesses, hantavirus cardiopulmonary syndrome (HCPS) and hemorrhagic fever with renal syndrome (HFRS). HCPS is caused by New World hantaviruses circulating in north America and south America. HFRS, a disease characterized by renal failure, hemorrhages, and shock with a case fatality of 0.1% to 10%, occurs primarily in Asia and Europe [1]–[3].

HFRS is highly endemic in mainland China accounting for 90% of the total cases reported in the world [6]. Although integrated intervention measures involving rodent control, environment management and vaccination have been implemented, HFRS remains a significant public health problem with more than 10,000 human cases diagnosed annually [7]. Hantaan virus (HTNV) and Seoul virus (SEOV) mainly carried by *Apodemus agrarius* (striped field mouse) and *Rattus norvegicus* (Norway rat), respectively, were known to be the crucial causative agents of HFRS in China [7], [8]. In addition, Amur virus (AMRV) and Puumala virus (PUUV) were detected recently from *Apodemus peninsulae* and *Clethrionomys glareolus* respectively in northeastern China [9], [10]. HFRS mainly occurred in rural area in the past. But recently, the endemic areas of the disease have extended from rural to urban areas and even to city centers [11].

Beijing metropolis is a newly affected region of HFRS, where the incidence of the disease has rapid increased since 1997 and the cases have been reported in all the 18 districts. The HFRS incidence seemed to be diverse considerably in difference places of Beijing according to the report from Beijing Center for Disease Control and Prevention (CDC). Previous epidemiological surveys revealed that hantaviruses detected in Beijing were all SEOV strains [12], [13], [14]. Although the environmental factors were related to the SEOV infectivity in rodent hosts and humans [15], [16], the hotspots of HFRS remained unclear and environmental factors weren't able to explain fully the distributional variation in incidence of disease in human.

The objectives of this study were to detect “hotspots” of HFRS in Beijing metropolis for effective control, to characterize variance of SEOV from the novel endemic region, and to investigate the possible association between SEOV genetic clusters and HFRS “hotspots”.

## Methods

### Ethics Statement

The research involving human materials was approved by the Ethical Review Board, Science and Technology Supervisory Committee at the Beijing Institute of Microbiology and Epidemiology. The informed consents were written by patients or their guardians and the related information was used anonymously. The research involving animal samples was approved by Animal Subjects Research Review Boards of the authors' institution and the study was conducted adhering to the institution's guidelines for animal husbandry.

### Data Collection and Spatial Scan Analysis

Records on HFRS cases reported in Beijing between 2004 and 2006 were obtained from the National Notifiable Disease Surveillance System (NNDSS). The vectorization of the village, street, and boundaries of each township was performed on a 1∶100,000 scale topographic map and digital map layers were created in ArcGIS 9.0 software (ESRI Inc., Redlands, CA, USA). Demographic information was integrated in terms of the administrative code. Each HFRS case was geo-coded according to their possible infected sites and a layer including information on HFRS cases was created and overlapped on the above digital map.

To identify the geo-spatial clusters i.e., “hotspots” with high HFRS risk of infection, the spatial scan statistic [17], [18] was performed by using SaTScan software [19]. The incidence rates of HFRS in 223 townships were calculated by using the fifth national census data in 2000. The maximum spatial cluster size was set to be 5% of the total population at risk and 9999 Monte Carlo replications were used to test the null hypothesis that the relative risk (RR) of HFRS was the same between any townships or their collection and remaining townships. A P-value<0.05 was considered statistically significant.

### RT-PCR and Phylogenetic Analysis

RNA extraction and reverse transcription reaction were performed as described previously [14]. The complete S sequences were generated from overlapping fragments by polymerase chain reaction (PCR) (primer pairs were presented in Table S1). Partial L sequences were generated using primer pairs previously described [20]. The sequences of SEOV strains were aligned using Clustalx1.8 software [21]. Phylogenetic trees were generated using the Bayesian Metropolis-Hastings Markov Chain Monte Carlo (MCMC) tree-sampling methods implemented by Mr. Bayes 3.1 software [22], using the GTR evolutionary model, with gamma- distributed rate variation across sites and a proportion of invariable sites. The run were stopped until the standard deviation of split frequencies was below 0.01. For comparison, phylogenetic analysis was also performed with the maximum- likelihood algorithm using Phylip software [23]. ML topologies were evaluated by bootstrap analysis of 100 ML iterations.

### Recombination Analysis

The automated suite of algorithms in the Recombination Detection Program 3 (RDP3) (available from http://darwin.uvigo.es/rdp/rdp.html) [24] was used to detect the recombination events. The programs included RDP, GENECONV, BootScan, MaxChi, Chimaera, SiScan, PhylPro, LARD, and 3Seq implemented in RDP3. According to the identified breakpoints, phylogenetic trees from spliced alignments were constructed by Bayesian methods using Mr. Bayes 3.1 software.

## Results

**Figure 1:**
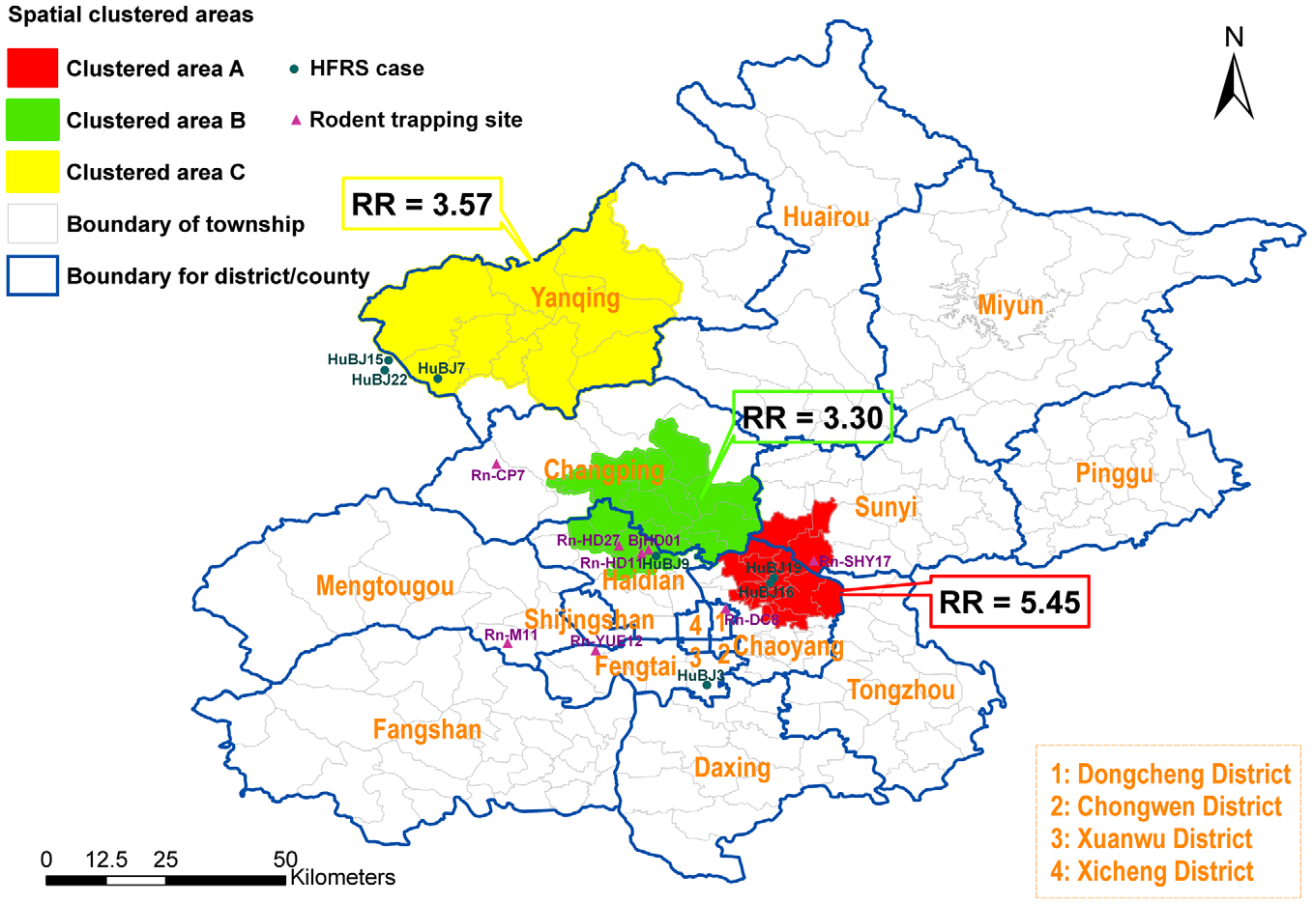
Spatial clustered areas with higher incidence of HFRS using spatial scan statistics. Spatial scan analysis was performed by moving windows statistics approach. It determined three statistically significant cluster areas (hotspots), designed as cluster area A with a Relative Risk (RR) of 5.45 (*P*<0.001) (the red area), cluster area B with RR of 3.57 (*P* = 0.002) (the yellow area) and cluster area C with RR of 3.3 (*P*<0.001) (the green area). The dots and triangles represent the sites from which sequence data are available, not all human cases or rodent captures in Beijing. The dots represented HFRS cases and the triangles represented the sites where the rodent hosts were captured. HuBJ15 and HuBJ22 were diagnosed in Beijing but their exposed sites fall outside of Beijing. They were included in the figure because they were in vicinity. HuBJ20 were diagnosed in Beijing but the exposed sites of the case were far away from Beijing, thus was not included in the figure.

A total of 322 confirmed HFRS cases were reported in Beijing metropolis from 2004 to 2006. The incident rates of HFRS in 223 townships ranged from 0/100,000 to 27/100,000. Three clustered areas (hotspots) were identified by spatial cluster analysis (Figure 1). The most likely clustered area, which was designated as clustered area A, contained 16 townships and located at the east of Beijing downtown, including a population of 570,000. The Relative Risk (RR) of the most likely clustered area was 5.45 (*P*<0.001). Clustered area B contained 14 townships, including a population of 650,000 with a RR of 3.57 (*P* = 0.002). This “hotspot” located at the northwest of Beijing and adjoined Hebei Province. Clustered area C contained 11 townships, including a population of 230,000 with a RR of 3.30 (*P*<0.001).

The entire S segment nucleotide sequences or partial L segment sequences of SEOV were obtained from 7 *R. Norvegicus* locating in different areas in Beijing (Rn-M11, Rn-DC8, Rn-YUE12, Rn-CP7, Rn-HD11, Rn-HD27, Rn-SHY17), 1 isolate (BjHD01) from *R. Norvegicus* and 8 HFRS patients (HuBJ3, HuBJ7, HuBJ9, HuBJ15, HuBJ16, HuBJ19, HuBJ20 and HuBJ22). The nucleotide sequences of the entire S segment of SEOV strains obtained from the HFRS patients and rodents had 97.0%–99.5% nucleotide sequences identity with each other and 95.0%–98.0% identity with those of other SEOV strains deposited in GenBank. 430-nucleotide L genomic sequences showed 97.4%–100% nucleotide sequence identity with each other and 93.7%–99.1% identity with those of other SEOV.

**Figure 2:**
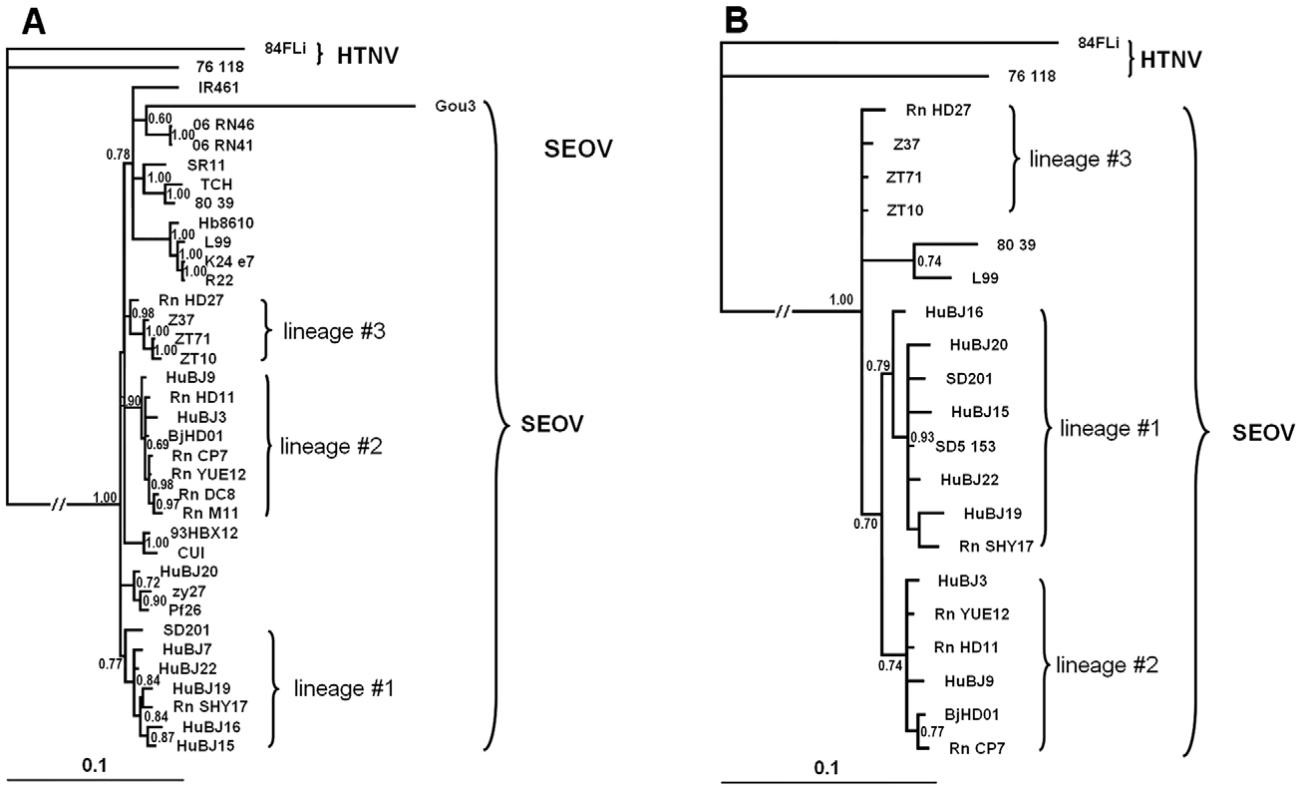
The Phylogenetic tree constructed by Mr. Bayes 3.1 software. It set the GTR evolutionary model with gamma-distributed rate variation across sites and a proportion of invariable sites. Numbers indicated posterior probalities for Bayesian inference. A: The phylogenetic tree based on the entire coding region of the 1,290-nucleotide S segment. B: The Phylogenetic tree based on 430-nucleotide L genomic sequence. Sequences from Genbank included 76–118 (M14626, NC_005222), IR461 (AF329388), SR11 (M34881), TCH (AF329389), 80-39 (NC-005236, X56492), Z37 (AF187082, AF285266), ZT10 (AY766368, EF581094 ), ZT71 (AY750171, EF190551), K24-e7 (AF288653), Gou3 (AB027522), Hb8610 (AF288643), R22 (AF488707), L99 (AF288299, AF288297), zy27 (AF406965), Pf26 (AY006465), 93HBX12 (EF192308). Sequences obtained in this study included CUI (GQ279395, ), SD201 GQ279385, HM748794), BjHD01 (AY627049, HM748802), HuBJ3 (GQ279391, HM748803), HuBJ7 (GQ279381), HuBJ9 (GQ279384, HM748795), HuBJ15 (GQ279390, HM748800), HuBJ16 (GQ279380, HM748804), HuBJ19 (GQ279389, HM748796), HuBJ20 (GQ279394, HM748805), HuBJ22 (GQ279379, HM748792), Rn-M11 (GQ279383), Rn-DC8 (GQ279386), Rn-YUE12(GQ279387, HM748801), Rn-CP7 (GQ279382, HM748793), Rn-HD11 (GQ279392, HM748798), Rn-HD27 (GQ279393, HM748799) and Rn-SHY17 (GQ279388, HM748791), SD5-153 (HM748797).

The phylogenetic tree based on entire coding region sequence of S segment showed that the strains circulating in Beijing clustered into three distinct lineages regardless of their host origins (Figure 2, Figure S1) (HuBJ20 was not included because it was obtained from a patient who got the disease in another place far away from Beijing and only was diagnosed in a hospital of Beijing). The phylogenetic tree based on complete S sequence showed the similar topology structure (data not shown). Five strains from HFRS patients clustered together with a rodent-originated sequences (Rn-SHY17), designated as #1. Two sequences from HFRS patients clustered together with six rodent-originated sequences (including BjHD01), designated as #2. One strain from a rodent (Rn-HD27) was distinct from other Beijing strains but close to those from Zhejiang Province where is more than 1, 000 km away from Beijing, which was designated as #3. The topology of the trees based on partial L sequences was similar to that based on S segment.

**Figure 3:**
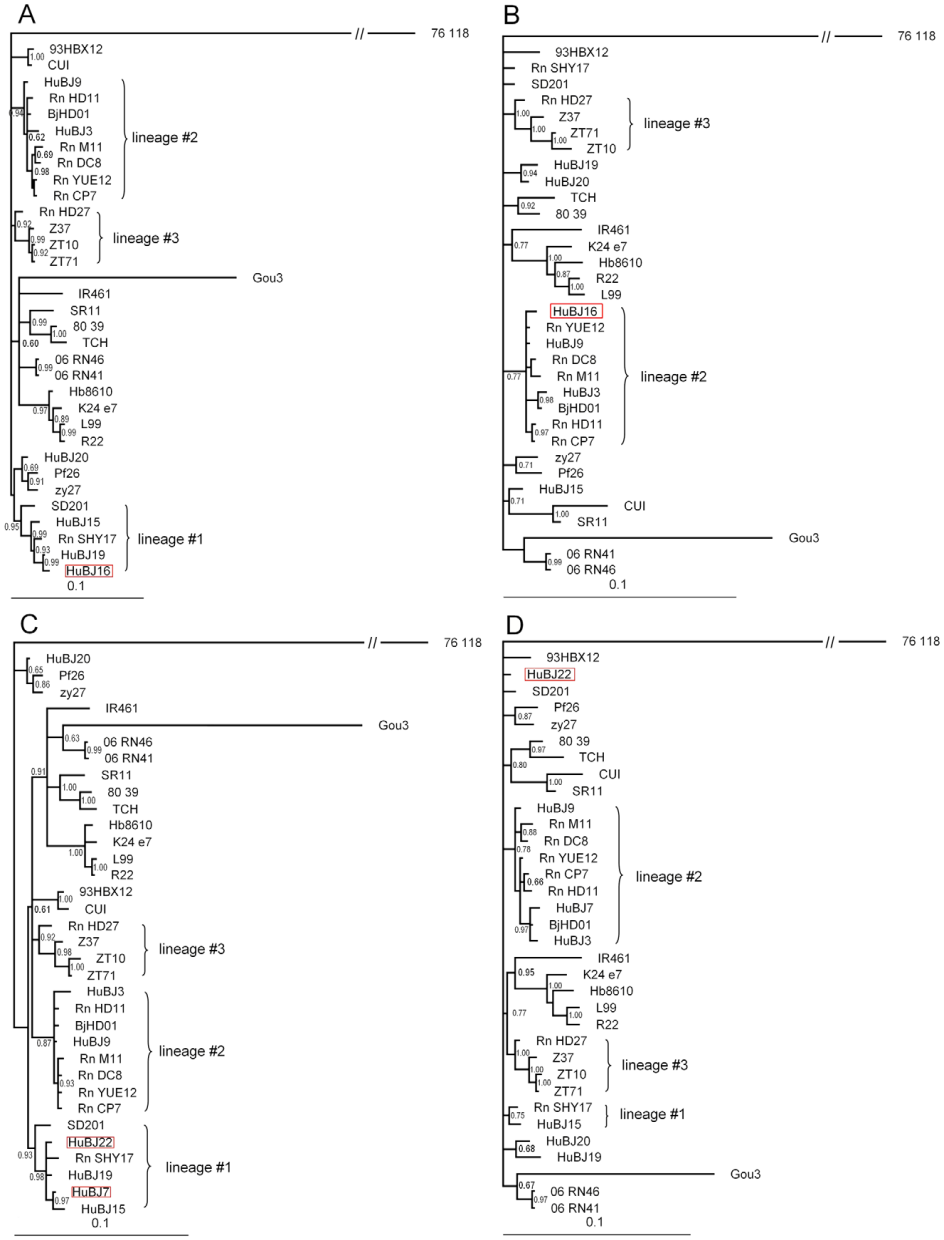
Phylogenetic trees from spliced alignments. Recombination event 1 involved the strain HuBJ16 and recombination event 2 involved the strain HuBJ22 and HuBJ7. A: Phylogenetic tree based on 116–1,020 nucleotide of the S segment. B: Phylogenetic tree based on concatenated sequence of the remainder of our sequence alignment. C: Phylogenetic tree based on 460–1,310 nucleotide of the S segment. D: Phylogenetic tree based on concatenated sequence of the remainder. Recombination daughter sequences were indicated in red pane.

In recombination analysis, three significant potential recombination events (PRE) were detected and two of them involved in the strains circulating in Beijing (Table 1). One of them involved HuBJ16 strain, whose major parent was Rn-YUE12 and minor parent was HuBJ19. Another PRE happened in HuBJ22 and HuBJ7 with HuBJ19 as the major parent and HuBJ3 as minor parent. In the Phylogenetic trees constructed using sequences of either the putative recombinant region or the region without recombination, changes in the topology of the trees could be observed (Figure 3). However, In the Phylogenetic trees according to the breakpoints of HuBJ22 strain, it was weakly supported, although the change in the topology of the trees could also be observed (data not shown). By contrast, no evidence of recombination was evident for the partial L segment sequence.

**Table 1:** Recombination Statistics of strains HuBJ16, HuBJ7 and HuBJ22.

Algorithm	HuBJ16		HuBJ7		HuBJ22	
	P-value	Breakpoint	P-value	Breakpoint	P-value	Breakpoint
MaxChi	1.885×10^−2^	114, 1031	8.490×10^−3^	444, 1315	8.490×10^−3^	566, 1408
Chimaera	2.138×10^−2^	113, 1031	1.318×10^−3^	459, 1311	1.318×10^−3^	–
SiScan	2.639×10^−5^	116, 1020	2.726×10^−4^	460, 1310	2.726×10^−4^	385, 1542
LARD	6.701×10^−5^	116, 1043	9.780×10^−3^	435, 1327	9.780×10^−3^	–
3Seq	5.457×10^−6^	114, 1031	2.931×10^−3^	460, 1310	2.931×10^−3^	385, 1408

The 3 spatial clustered areas of HFRS seemed associated with different SEOV strains. All SEOV strains in lineage #1 were from clustered area A and clustered area B, although the two clustered areas were not contiguous to each other. Apart from HuBJ15, four patient-originated strains and one rodent-originated strain had a special homologous substitution of *asparagine* to *threonine* at position 259 of the deduced amino acid sequences of the N protein, which was distinct from all other SEOV strains. Interestingly, all the strains involving PRE belonged to lineage #1. Most strains from rodent hosts were in lineage #2 and scattered in different areas of Beijing. Among them, four rodent-originated strains (Rn-M11, Rn-DC8, Rn-YUE12 and Rn-CP7) had a same special homologous substitution of *asparagine* to *serine* at amino acid position 214, which had never been detected from any other SEOV strains. None of the 4 strains were found in any spatial clustered areas with high RR value. Strains without the homologous substitution at position 214 in lineage #2 could be divided into two parts. One strain from a patient (HuBJ 9) and two strains (Rn-HD11, BjHD01) from the rodent hosts located in clustered area C. Another strain (HuBJ3) from a patient located in southwest of Beijing, an area with low RR value. Rn-HD27 strain in lineage #3 without any special substitution also located in clustered area C.

## Discussion

Cluster analyses are important in epidemiology in order to detect aggregation of disease cases, to test the occurrence of any statistically significant clusters, and ultimately to find evidences of etiologic factors. Cluster analysis identifies whether geographically grouped cases of disease can be explained by chance or are statistically significant. Spatial scan statistic [17], [18] implemented in SaTScan software [19] is being widely used to detect the clusters of many infectious diseases [25], [26]. Recently, we analyzed the distribution of HFRS cases nationwide using GIS-based spatial analysis and highlight geographic areas where the population had a high risk of acquiring the disease [27]. Beijing is one of the emerging endemic areas of HFRS in recent years. In this study, the district-based digital map layers and three-year surveillance data were analyzed altogether and a moving-window scan statistics approach were used to determine the geo-spatial “hotspots”. It reduced the effects resulted from probable reporting bias and selecting bias. The results of the study suggested that there were three spatial “hotspots” of HFRS in Beijing, where the population had a high risk of acquiring the disease and intervention should be enhanced.

Mice of species *Apodemus agrarius* are quite close to people in rural areas. Consequently, HTNV is still the major cause of HFRS in China. However, more and more HFRS patients caused by SEOV were reported in mainland China. It is possibly related to its animal host, *R. Norvegicus*, which is spatially more close to human beings than any hosts of other hantaviruses. Sometimes, they can even migrate to the places far away by traveling on vehicles such as ship, train or truck [4]. They may cause international transmission by carrying HV to another place, just as presumed in Taiwan and Indonesia [28], [29]. Beijing is the capital of China, transportation of goods and human migration with other regions that followed the rapid economic development was quite frequent in recent years. It is not surprising that several lineage of SEOV were circulating simultaneously in Beijing and appearance of recombination.

It was reported that prevalence of SEOV in rodents was different among districts in Beijing [30]. However, it was not consistent with clustered areas based on GIS-based spatial analysis, suggesting other factors might be related to the incidence of the disease in human.

Incorporating the additional genetically related taxa into a typical analysis usually leads to better phylogenetic resolution and comprehensive understanding. It had reported the phylogenetic tree based on partial M segment of most strains from rodents in Beijng (30). Since the amount of available biological material from patients was limited, we proceeded to amplify and sequence the entire S genome segment and partial L segment.

The S-segment phylogenetic tree indicated that at least 3 lineages of SEOV were circulating in Beijing (figure 2). Strains in the first lineage coincided with the spatial clustered areas of HFRS with high RR values and most strains had special substitution of *asparagine* to *threonine* at amino acid position 259 of the N protein. All the strains involving recombination signals in Beijing were all in lineage #1 and located in HFRS clustered areas with the highest RR value. It seemed that the PRE was not a seldom event in these areas since more than one PRE were detected. Our data, together with a number of other studies, suggested that homologous recombination events might be not an uncommon process in hantavirus natural evolution [31]–[33]. In lineage #2, some strains had substitution of *asparagine* to *serine* at position 214 of the N protein. All of them could only be detected from rodent hosts and located in areas with lower risk of HFRS occurrence. The relationship between the hantavirus lineages and the cluster areas could be observed in partial L-segment phylogenentic tree. When compared with previous study based on partial M segment, there was another lineage circulating in Beijing (30). Moreover, the relationship between the hantavirus lineages and the cluster areas could also be observed except that two strains distributed in areas with lower risk of HFRS (Dongcheng district) seemed fall into the lineage #1 (30). It should be noted that the captured sites of the two rodents were in a railway station and were closed with the clustered area A, which might be the reason for the two strains different from strains in areas with low risk of HFRS but clustered together with strains in lineage #1. Longer sequence such as complete S segment analysis of the two strains might be helpful to elucidate their phylogeny and genetic characteristic. Unfortunately, the limited amount of available biological materials from the two rodents hindered us to complete the task.

Our findings, together with previous study, indicated that several lineage of SEOV were circulating simultaneously in Beijing. Furthermore, it suggested that genetic characteristics of SEOV might be associated with some risk areas of HFRS in Beijing. This correlation could be that different strains circulating in different areas were evolving at a different rate or different pattern due to environmental diversity, hence accumulated different mutations. On the other hand, mutations and recombination might play an important role for SEOV to adapt to hosts in an emerging endemic area such as Beijing.

It seemed that the mutations at amino acid position 214 and 259 of the N protein didn't happened by chance, since the same mutations happened in more than one strains from several areas and these mutations seemed to consistent with the spatial cluster areas. Compared with other hantaviruses, the nucleotide sequences of SEOV seemed to be more conservative. The S genome segment nucleotide sequences identity of SEOV ranged from 93.7% to 99.5% and amino acid sequences identity ranged from 96.6% to 100%. Although there were some evidences that the two observed mutations located in regions with the important function to N protein for some hantaviruses [34]–[37], laboratorial data about the effect of the mutation to the biological function of S segment gene products were absent at present. This issue needs to be further evaluated.

## Supporting Information

Alternative Language Abstract S1 Chinese abstract translated by author Shu-Qing Zuo.
(0.02 MB DOC)

Figure S1 Phylogenetic trees constructed by maximum-likelihood algorithm. A: Phylogenetic tree based on entire encoding region sequence of S segment. B: Phylogenetic tree based on partial L segment sequence. Only bootstrap values greater than 70% were shown.
(0.25 MB TIF)

Table S1 The primers pairs for amplification of complete S sequence.
(0.04 MB DOC)
