# Supplementary material for: Geo-spatial Hotspots of Hemorrhagic Fever with Renal Syndrome and Genetic Characterization of Seoul Variants in Beijing, China
Source: PLoS Negl Trop Dis. 2011 Jan 11;5(1):e945. doi: 10.1371/journal.pntd.0000945 (PMC3019113; doi:10.1371/journal.pntd.0000945)
Supplement: Alternative Language Abstract S1 — Chinese abstract translated by author Shu-Qing Zuo. (0.02 MB DOC) [file pntd.0000945.s001.doc]

# 中国北京地区肾综合症出血热“地理-空间热点”与Seoul病毒基因变异研究

**摘要**

***背景：*** 在中国大陆，肾综合症出血热是一种严重的自然疫源性疾病。近年来，该病呈现出向城市地区特别是中心城市蔓延的趋势。北京地区即为近年来新出现的流行地区之一。但该地区不同区县的发病率似乎并不完全相同。

***方法/主要发现:*** 根据北京地区2004年至2006年的肾综合症出血热被动监测数据，采用基于地理信息系统的空间扫描分析确定北京地区存在3个肾综合症出血热的地理聚集区或称“地理-空间热点”。这三个“热点”的相对危险度分别为5.45, 3.57 和3.30；对同一时期在北京不同地区采集的来自不同宿主和病例标本的 Seoul病毒S片段编码区序列和L片段部分序列进行系统发育分析，提示北京地区流行的Seoul病毒至少可以分为三个小的进化分支，应用RDP3软件和比较系统发育分析能检测到第一进化支的Seoul病毒存在两个潜在的重组事件。此外，研究发现不同的小进化分支中部分病毒的N蛋白具有不同的氨基酸变异，而且不同进化来源的病毒以及具有不同氨基酸变异特征的病毒分布与具有不同相对危险度的地理聚集区的分布具有一定的相关关系。

***结论/意义:***  发现北京地区肾综合症出血热病例分布不均衡，存在几个“地理-空间热点”，今后应重点加强这些地区的防控工作。Seoul 病毒的变异和重组可能与该病在北京地区的分布“热点”相关，尚需进一步研究和证实。
