# Supplementary figures and images for: Geo-spatial Hotspots of Hemorrhagic Fever with Renal Syndrome and Genetic Characterization of Seoul Variants in Beijing, China
Source: PLoS Negl Trop Dis. 2011 Jan 11;5(1):e945. doi: 10.1371/journal.pntd.0000945 (PMC3019113; doi:10.1371/journal.pntd.0000945)

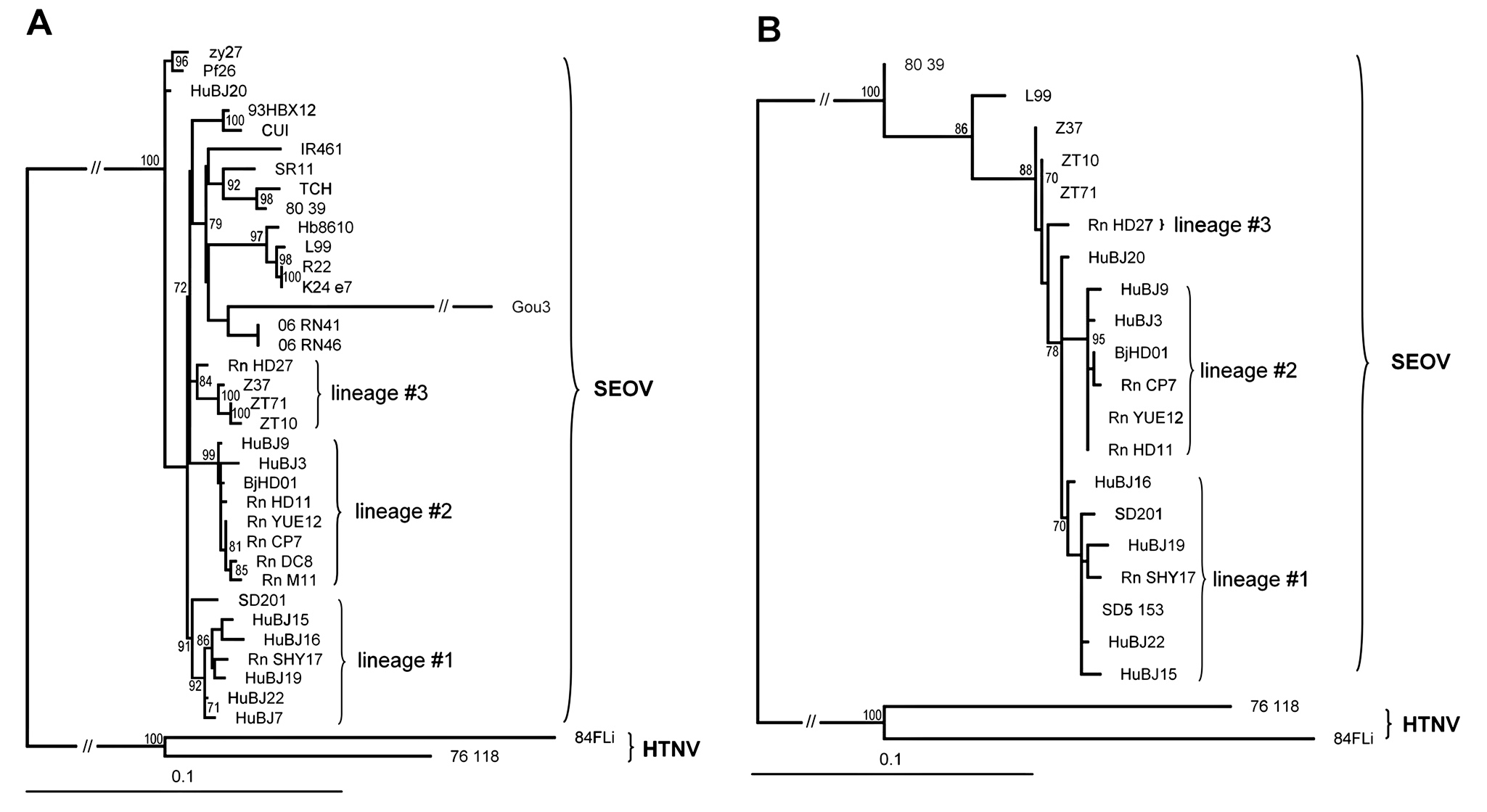

Supplement: Figure S1 — Phylogenetic trees constructed by maximum-likelihood algorithm. A: Phylogenetic tree based on entire encoding region sequence of S segment. B: Phylogenetic tree based on partial L segment sequence. Only bootstrap values greater than 70% were shown. (0.25 MB TIF) [file pntd.0000945.s002.tif]
