# Supplementary material for: Geo-spatial Hotspots of Hemorrhagic Fever with Renal Syndrome and Genetic Characterization of Seoul Variants in Beijing, China
Source: PLoS Negl Trop Dis. 2011 Jan 11;5(1):e945. doi: 10.1371/journal.pntd.0000945 (PMC3019113; doi:10.1371/journal.pntd.0000945)
Supplement: Table S1 — The primers pairs for amplification of complete S sequence. (0.04 MB DOC) [file pntd.0000945.s003.doc]

Table S1. The primers pairs for amplification of complete S sequence

| Primers | Primer sequence (5’-3’) | Polarity |
| --- | --- | --- |
| SF1 | TAGTAGTAGACTCCCTAAAGA | + |
| SR616 | GAATCTTCCAGGTGTTATCTC | - |
| SR670 | TACCATGTTCCTTGCCTTTAT | - |
| SF424 | TCATTYGTGGTCCCRATCATCTT | + |
| SF812 | CTGGGAATCCTGTRAATCGTG | + |
| SR1008 | CCTAAYTCAGCCATCCCTCCG | - |
| SF1125 | CTAYCAATCATACCTCAGACGC | + |
| SR1307 | ATARTTTCATAGGTTCCTGGTTWG | - |
| SR1745 | TAGTAGTAGTATGCTCCCTAAAAAGACA | - |
